# Abnormal cortisol profile during psychosocial stress among patients with schizophrenia in a Chinese population

**DOI:** 10.1038/s41598-022-20808-1

**Published:** 2022-11-03

**Authors:** Xiaoyu Zhu, Yu Zhu, Junchao Huang, Yanfang Zhou, Jinghui Tong, Ping Zhang, Xingguang Luo, Song Chen, Baopeng Tian, Shuping Tan, Zhiren Wang, Xiaole Han, Li Tian, Chiang-Shan R. Li, L. Elliot Hong, Yunlong Tan

**Affiliations:** 1 Peking University HuiLongGuan Clinical Medical School, Beijing HuiLongGuan Hospital, Beijing, People’s Republic of China; 2 Department of Psychiatry, Yale University School of Medicine, New Haven, CT USA; 3 Department of Physiology, Faculty of Medicine, Institute of Biomedicine and Translational Medicine, University of Tartu, Tartu, Estonia; 4 Department of Psychiatry, Maryland Psychiatric Research Center, University of Maryland School of Medicine, Baltimore, USA

**Keywords:** Schizophrenia, Diagnostic markers

## Abstract

Accumulating evidence suggests that hypothalamic-pituitary-adrenal axis dysfunction might play an important role in the pathophysiology of schizophrenia. The aim of this study was to explore the cortisol response to psychological stress in patients with schizophrenia. In this study, patients with schizophrenia (n = 104) and healthy volunteers (n = 59) were asked to complete psychological stress challenge tasks, which included the Paced Auditory Serial Addition Task and Mirror-Tracing Persistence Task, and pre- and post-task saliva samples were collected to measure cortisol levels. Emotions and psychopathology were assessed by the Positive and Negative Affect Schedule and Positive and Negative Syndrome Scale. The results showed (1) that the cortisol response and negative emotions in patients with schizophrenia differed significantly from those in healthy volunteers, (2) there were significant interactions between the sampling time and diagnosis for saliva cortisol levels, (3) there were significant interactions between the scoring time and diagnosis for the negative affect score of the PANAS, and (4) the changes in salivary cortisol levels and negative affect scores before and after the psychological stress challenge tasks were not correlated with clinical symptoms in patients with schizophrenia. These findings indicated an abnormal cortisol profile in patients with schizophrenia, which might be a biological characteristic of the disease.

## Introduction

Schizophrenia is a severe neuropsychiatric disorder for which the etiology remains unclear; however, the interaction of multiple biological and environmental factors plays an important role in its pathophysiology. Several models of stress-vulnerability have been proposed to better understand the relationships among vulnerability, stress, and schizophrenia^1–4^. The hypothalamic-pituitary-adrenal (HPA) axis is one of the primary systems that moderate the physiological response to psychological and physiological stressors^5^, and its function can be assessed by measuring cortisol secretion levels.

Cortisol can be detected in tissues such as hair and nails—reflecting chronic secretion patterns—and in body fluids (saliva, blood, and urine), which are more suitable to detect acute levels^6^. The Cortisol Assessment Checklist is a widely accepted metric for assessing cortisol levels, such as basal cortisol level, cortisol awakening response (CAR), and reactive cortisol level^6^. The basal cortisol level reflects the fluctuation of the HPA axis throughout the day and is often assessed using twenty-four-hour urinary cortisol^7^. Conversely, CAR measures the processes of the rapid rise and peak of cortisol levels within 30–60 minutes after awakening in the morning^8^. The cortisol stress response is a measure of HPA axis activation following exposure to a stressor and is characterized by (1) the sympathetic nervous system activation, (2) subsequent activation of the HPA axis, and (3) increased cortisol secretion^9^. The effective cortisol response captures the rapid rise in cortisol levels a few minutes after exposure and peak at around 30 minutes, which is followed by a rapid decline^10,11^. This physiological process plays a key role in the body’s ability to adapt to the environment and respond to threats^12^. The cortisol stress response is an important indicator of HPA axis function. However, although CAR mainly reflects arousal, it is also affected by the stress level of the previous day^8,13,14^. In this way, both CAR and cortisol stress response can be said to reflect the mobilization and rehabilitation capability of the HPA axis.

The association between cortisol and schizophrenia has been widely recognized^12^. In general, patients with schizophrenia have abnormally elevated cortisol levels, but the results of current studies are inconsistent^12,15–17^. A recent meta-analysis pointed out that aberrant morning cortisol levels measured peripherally (using blood or saliva) were observed more frequently in patients with schizophrenia than in controls; however, the results might have been influenced by factors such as the illness stage, mood state, and psychotropic medication^15^. Some studies have also noted a blunted CAR in patients with schizophrenia^18–20^. Although antipsychotic treatment can alleviate the effects of abnormal diurnal cortisol levels^4,21^, the blunted CAR persists^22^. These results suggest that changes in cortisol levels might be a more stable indicator.

Other studies also found heightened cortisol secretion or flattened CAR among individuals who could not be diagnosed with schizophrenia but who met the clinical high-risk (CHR) criteria for psychosis when compared to that in healthy controls^13,23–26^. Similar results have been reported among relatives of patients with schizophrenia^25–27^. These findings suggest that abnormal cortisol secretion predates the onset of psychiatric symptoms and may be genetic in nature. Cortisol-related abnormalities and the HPA axis dysfunction that they signify have yet to be identified as predictors for the occurrence and development of schizophrenia; however, more research should be performed, given their potential value as biomarkers.

Although blunted CAR may be a stable indicator, its measurement time is a limitation and requires a high degree of patient cooperation.

In addition to CAR, another indicator of changes in cortisol levels is cortisol stress response. The cortisol stress response can be stimulated by a more flexible manual psychological stress test. Therefore, we sought to explore whether the cortisol stress response has a clinical significance similar to that of CAR. We speculated that the dysregulation of the HPA axis mobilization in patients with schizophrenia is also reflected in the cortisol stress response and that this change is independent of clinical symptoms, similar to the blunting phenomenon of CAR.

The cortisol response to psychosocial stress is observed but blunted in patients with schizophrenia when compared to that in controls^12^. Girshkin et al. reported that patients with schizophrenia did not show any cortisol increase in response to magnetic resonance imaging (MRI) as a stressor^17^, unlike healthy individuals^28^. This differed from the cortisol stress response shown among patients with schizophrenia in most studies using laboratory-based psychological stress challenge tasks^12,16^. Although the cause for the differences in cortisol stress response toward different stressors has not yet been elucidated, one possible explanation for this was that stress during an MRI scan mainly involves the passive perception of noise, which may be attenuated by apathy to the environment caused by schizophrenia. However, although different stressors lead to different outcomes in studies, differences in cortisol stress response are pervasive in patients with schizophrenia and controls.

In this study, we aimed to investigate the difference in cortisol response to psychological stress between individuals with schizophrenia and healthy controls in the Chinese population and to test the hypothesis that patients with schizophrenia have an abnormal cortisol response, which represents dysregulated HPA function, and that HPA axis dysfunction has the potential to serve as a biomarker for schizophrenia. Salivary cortisol secretion is a reliable noninvasive indicator of free cortisol in plasma and has been used as an index for HPA axis activity in many studies^29^; thus, we examined cortisol levels by testing saliva collected before and after a laboratory-based psychological stress challenge task.

## Material and methods

### Participants

Patients were recruited from inpatients or outpatients at the Beijing HuiLongGuan Hospital from 2017 to 2019. The inclusion criteria for the patients were as follows: (1) diagnoses confirmed according to the Structured Clinical Interview in the Diagnostic and Statistical Manual of Mental Disorders (DSM)-IV diagnostic criteria for schizophrenia, (2) age between 18 and 60 years, and (3) Han Chinese ancestry.

Patients were excluded if they had (1) evidence of a significant physical illness or neurological condition, (2) substance dependence or current substance abuse (except nicotine) at the time of screening, (3) a psychiatric disorder other than schizophrenia according to DSM-IV criteria, (4) any endocrine disorder, or (5) received any immunotherapies.

Age- and sex-matched healthy volunteers were recruited from the local community. Healthy volunteers were excluded if they had (1) evidence of a significant physical illness or neurological condition, (2) current and ongoing substance abuse (except nicotine) at the time of screening, (3) any psychiatric disorder according to the DSM-IV criteria, or (4) any endocrine disorder or (5) received any immunotherapies.

### Psychological stress challenge task

We used a psychological stress challenge task as a stressor and to assess the capability of the participants to persist in goal-directed behavior while under stress. The task consisted of two tests, each lasting for 7 minutes: the Paced Auditory Serial Addition Task (PASAT) and the Mirror-Tracing Persistence Task (MTPT), both of which have been applied in psychiatric research^30^. During the PASAT, the participants were asked to add multiple numbers that were displayed sequentially on a screen and received a noise penalty upon a miscalculation. During the MTPT, the participants were asked to trace the outline of a star on the screen using a mouse with the cursor moving in the opposite direction to the hand. If the cursor lost track or was fixed, it was judged as an error, causing annoying noises, and the drawing process was reset. The computer program adjusted the number display speed and the width of the star according to the participant’s performance to ensure the task was challenging for everyone.

As these two tasks involve two different skills (computing and hand-eye coordination), their combination helps avoid biases that may be caused by individual expertise and is widely used to assess distress intolerance^16,30–32^. The two tasks were performed sequentially and in a random order. The whole process was completed in the form of human-computer dialogues, thus avoiding the subjective influence of researchers. The participants completed the psychological stress tasks in different rooms or at different times, so as to avoid interfering with each other. During the tasks, staff were nearby to instruct all participants without interfering.

The participants could opt-out at any time during the task. They were offered 40 Yuan for completing two tasks and 20 Yuan for failing to complete one or both tasks.

We sought to assess the participants’ ability to adhere to goal-directed behavior under pressure and to judge their distress tolerance. According to the paradigm of previous research, we determined distress tolerance (DT) when participants persisted in completing either of the tasks, and we determined distress intolerance (DI) when participants failed to complete both tasks^16,30–32^.

### Saliva collection and processing

To reduce the effects of cortisol-level changes over time, all tasks and saliva sample collection were performed between 12:00 and 16:00. Afternoon samples at this time reflect a more stable cortisol level within the diurnal rhythm, show less fluctuation and lower cortisol concentrations than observed during the morning post awakening hours, so that stress responses are less affected. Therefore, this period is commonly chosen in studies that are aimed to measure the cortisol stress response^16,31^. Because of the difficulty of the psychological stress challenge task, participants experienced stress during the task. The process of avoiding mistakes and, therefore, the noise penalty made participants more cautious, further increasing stress. Considering that cortisol levels usually start to rise a few minutes after stressor exposure and peaks at around 30 minutes^10,11^, we collected three saliva samples from each participant: before starting the tasks and after resting for 20 and 40 minutes following the tasks. To reduce any confounders, all participants were asked not to do strenuous exercise, eat, drink, smoke, or brush their teeth for 1 hour before the test, and then to rinse their mouths with water and spit out 10 minutes before the test to reduce food residues in the mouth.

Saliva collection was performed either by reducing tongue movement and directly collecting saliva or by letting participants chew on a cotton swab to stimulate salivary gland secretion—the participants were asked to chew gently to avoid blood leaking onto the swab. As we aimed to collect saliva samples three times in a short period, the amount of saliva collected by the direct method would gradually decrease, rendering the third sample inadequate to meet the detection requirements. Therefore, the chewing collection method was used.

Saliva samples were collected using a Salivette device (Sarstedt AG & Co Kommanditgesellschaft SarstedtstraBe 1 51588, Nümbrecht, Germany). The participants were asked to chew the swabs thoroughly and gently for 2 minutes at each sample collection time to make sure more than 1 mL of saliva was collected. The saliva sample was then frozen at -20°C for 24 hours. After thawing the frozen saliva, it was centrifuged at 4°C for 10 minutes (4000 rpm) to obtain more than 0.5 mL saliva from each sample. Then, the processed saliva sample was placed into a centrifuge tube; the internal standard solution was added, followed by the precipitant; and the tube was vortexed and centrifuged for 10 minutes. The supernatant was then taken out and blown dry with nitrogen. A derivatization reagent was added to the dried sample, and the sample was placed in the oven for derivatization. After derivatization, the supernatant was used for liquid chromatography, and proteins were quantified via high-performance liquid chromatography/tandem mass spectrometry, using standard protocols ^33^. The intra-trial coefficients of variation for high, medium, and low quality of salivary cortisol were 8.8%, 5.6%, and 10.1%, respectively.

### Clinical assessment

All participants completed the Positive and Negative Affect Schedule (PANAS)^34^ before the start of the first task, after completing (or aborting) the first task, and after completing (or aborting) the second task. This scale contains ten positive affect and ten negative affect (NA) items. Considering that the stress response mainly involves negative emotions, we only used the NA scores.

**Figure 1:**
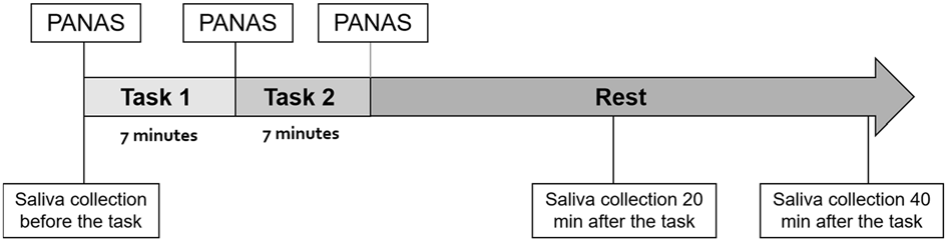
Outline of the experimental test.

A total of 176 participants, including 112 patients with schizophrenia and 64 healthy volunteers, completed the psychological stress challenge task, PANAS, and saliva collection. Psychopathology was assessed by an attending psychiatrist using the Positive and Negative Syndrome Scale (PANSS)^35^. Our psychiatric examinations were conducted using the structured clinical interview for the PANSS (SCI-PANSS), a formatted interview tool, and PANSS was scored based on the content of the interview tool. The process is shown in Fig. 1.

### Statistical analyses

The saliva cortisol data collected in the patient and control groups were analyzed. Data that deviated from the 25–75% range for each set in each group of data by more than three times the interquartile range (IQR) of the set were defined as extreme values and excluded from the final analysis. Finally, the data of 104 patients and 59 controls were included.

Salivary cortisol level and PANAS score data showed approximately normal distribution and are presented as the mean and standard deviation. First, a repeated-measures analysis of variance (ANOVA) was performed on salivary cortisol levels and PANAS scores to examine the interaction between multiple factors, such as diagnosis (patients or controls), sampling time (before and 20 and 40 minutes after testing), and distress intolerance (yes or no). Then a one-way ANOVA was used to examine the impacts of each factor on the variables if the interaction existed. The Mann–Whitney U and Wilcoxon signed-rank tests were used to examine between-group differences in age, PASAT error rate, MTPT error rate, and years of education. The chi-squared test was used to examine between-group differences in sex, distress intolerance and smoking rates. The significance level was set at 5% (*p* < 0.05).

### Ethical statement

This research follows the 1964 Declaration of Helsinki and subsequent amendments or similar ethical standards. This research was approved by the Human Research Ethics Committee of Beijing HuiLongGuan Hospital [Protocol No. 2017-49]. Written informed consent was obtained from all participants.

## Results

### Participant characteristics

In terms of age, sex, smoking status, education level, and PASAT error rate, there were no significant differences between the patients with schizophrenia and the healthy controls. The error rate of MTPT in the patients with schizophrenia was significantly higher than that of the controls. The proportion of participants with distress intolerance was significantly higher in the patients than in the healthy controls (Table 1).

**Table 1:** Participant demographics and clinical characteristics

	Patients (*n*=104)	Healthy controls (*n*=59)	Statistics (z/χ^2^)	*P* value
Age (years, mean ± SD)	40.43±12.67	37.42±12.72	− 1.444	0.149
Sex (Male %)	59 (56.7%)	34 (57.6%)	0.012	0.912
Distress intolerance (DI %)	18 (17.3%)	1 (1.7%)	8.911	0.003
PASAT error rate (mean ± SD)	0.161±0.132	0.149±0.109	− 1.720	0.085
MTPT error rate (mean ± SD)	0.712±0.288	0.309±0.199	− 8.148	<0.001
Smoking status (smokers %)	32 (30.8%)	13 (22.0%)	1.437	0.231
Education (years, mean ± SD)	12.63±3.75	13.08±2.57	0.492	0.643

*PASAT* Paced auditory serial addition task, *MTPT* Mirror-tracing persistence task.

### Analyses of cortisol levels

We found no significant effect of interaction between sampling time and sex (F = 0.032, *p* = 0.951) or sampling time and smoking status (F = 0.350, *p* = 0.669) on cortisol levels. The main effects of both sex (F = 3.348, *p* = 0.069) and smoking status (F = 0.368, *p* = 0.545) were not significant. Using age as a covariate, the interaction and main effects were still not significant (all *p*-values > 0.05).

For ease of description, we assigned subscripts to the variables of salivary cortisol data collected at different times: 1 represented data collected before the task, 2 represented data collected 20 minutes after the task, and 3 represented data collected 40 minutes after the task.

**Figure 2:**
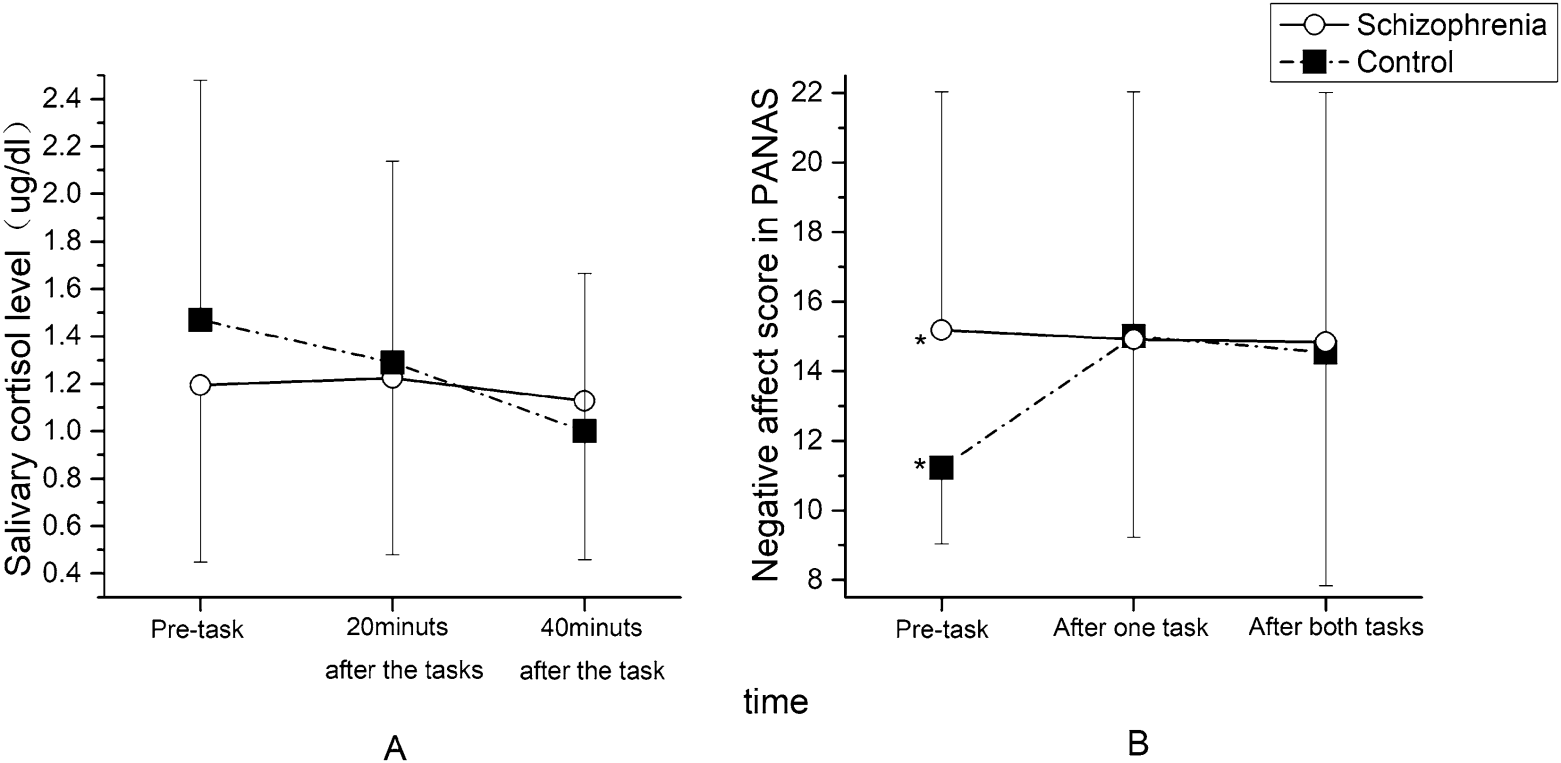
Salivary cortisol levels **(A)** and NA scores **(B)** of patients and controls (*p < 0.05).

In the patient group, although saliva cortisol levels tended to rise first and then fall, the change was not significant (*p* > 0.05). In the control group, saliva cortisol levels were the highest before the task and then gradually decreased (F = 13.383, *p* < 0.001) (Fig. 2A).

There was a significant interaction between sampling time and diagnosis (F = 6.961, *p* = 0.002). To exclude the influence from the interaction, we used one-way ANOVA to examine the impacts of sampling time and diagnosis on the salivary cortisol levels of the two groups separately. Before the task, the saliva cortisol level of the control group tended to be higher than that of the patient group (F_1_ = 3.875, *p*_1_ = 0.051), but there were no significant differences between the two groups 20 minutes (F_2_ = 0.261, *p*_2_ = 0.610) or 40 minutes (F_3_ = 1.361, *p*_3_ = 0.245) after the task. The main effect of sampling time on the patient group was not significant (F = 1.250, *p* = 0.287), while the main effect of sampling time on the control group was significant (F = 13.383, *p* < 0.001). This indicated that the stress response of cortisol during the psychological stress challenge task test was blunted among patients with schizophrenia and more active among the healthy controls.

### NA scores

For the ease of description, we assigned subscripts to the variables of NA scores evaluated at different times: 1 represented data before the task, 2 represented data after one task, and 3 represented data after both tasks.

There was a significant effect of interaction between scoring time and diagnosis on NA scores (F = 14.744, *p* < 0.001). To exclude the influence from the interaction, we used one-way ANOVA to examine the impacts of scoring time and diagnosis on the NA scores of the two groups separately. The NA scores of the patients with schizophrenia were significantly higher before the task (F_1_ = 18.442, *p*_1_ < 0.001) than those of the controls. There were no significant differences between patients and controls in the NA scores after completing one task (F_2_ = 0.006, *p*_2_ = 0.937) and in the NA scores after completing two tasks (F_3_ = 0.066, *p*_3_ = 0.798). The main effect of scoring time in the patient group was not significant (F = 0.307, *p* = 0.712), while the main effect of scoring time in the control group was significant (F = 16.168, *p* < 0.001). These results indicated that patients with schizophrenia had more negative emotions before the test, while the psychological stress challenge tasks had no significant effect on this experience. The controls had fewer negative emotions before the test, and their negative emotions increased during the test (Fig. 2B).

### Distress tolerance and intolerance

**Figure 3:**
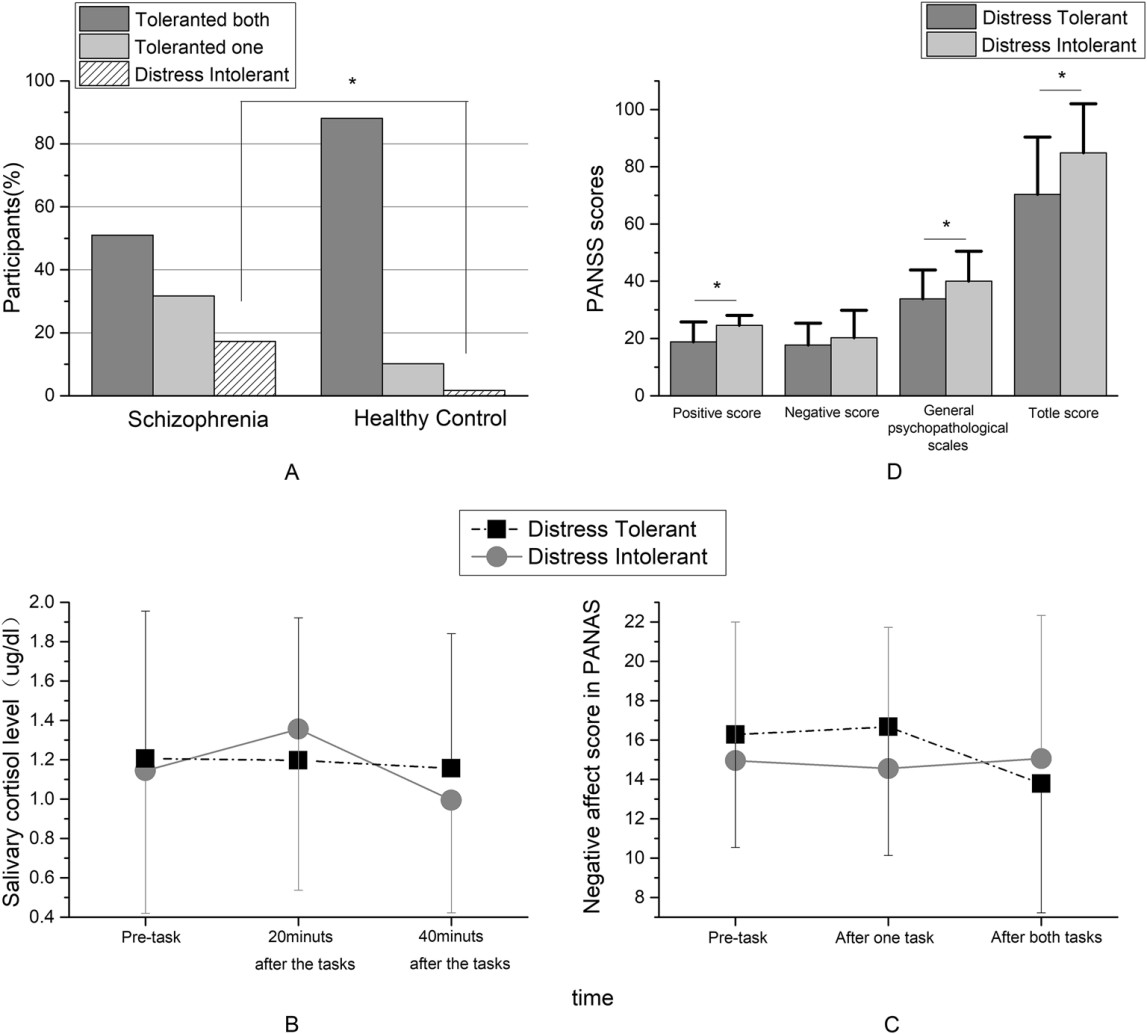
Analysis related to distress tolerance (*p< 0.05). (**A**: comparison of task completion; **B**: comparison of salivary cortisol levels; **C**: comparison of NA scores; **D**: comparison of PANSS scores).

In the control group, most participants persisted in completing the task, and only one participants chose to drop out (showed distress intolerance). The rate of distress intolerance in the schizophrenia group was significantly higher (Fig. 3A).

As there was only one participant who showed distress intolerance in the control group, further grouping and comparison were not necessary. Therefore, we only divided the patients with schizophrenia into subgroups based on stress tolerance and compared the subgroups.

Although the trends of cortisol-level changes differed between the subgroups, there was no significant interaction between sampling time and tolerance (F = 1.985, *p* = 0.145), and the main effects of sampling time or tolerance were not significant (F = 2.991, *p* = 0.058 and F = 0.017, *p* = 0.897, respectively) (Fig. 3B).

In the NA scores, there was a significant interaction between scoring time and tolerance (F = 4.338, *p* = 0.019), but the main effects of scoring time (F = 2.604, *p* = 0.083) and tolerance (F = 0.180, *p* = 0.672) were not significant (Fig. 3C). We performed a post-hoc one-way ANOVA to compare the scores of the DI and DT groups at different scoring times and found no significant differences (all *p*-values > 0.05).

### Clinical symptoms

The PANSS positive subscale, general psychopathological subscale, and total scores differed significantly between patients who showed distress tolerance and those who did not. A higher score for the distress-intolerance group indicated that these patients’ symptoms may be more severe (Fig. 3D).

We performed a correlation analysis of the total PANSS, positive subscale, negative subscale, general psychopathological subscale, and NA scores, as well as salivary cortisol levels of the patients with schizophrenia. In the correlation test between the total PANSS scores and cortisol levels, the significance level was *p* < 0.0167 (0.05/3) after Bonferroni correction. The correlation test between the PANSS subscale scores and cortisol levels resulted in a significance level of *p* < 0.0056 (0.05/9) after Bonferroni correction. In the correlation test between the NA scores and cortisol levels, the significance level was *p* < 0.0056 (0.05/9) after Bonferroni correction. Most of the data showed no significant correlations. The cortisol levels 20 minutes after the task were correlated with negative subscale scores (r = 0.277, *p* = 0.004). However, the correlation is generally considered meaningful only if the correlation coefficient is greater than 0.3; the above correlation coefficients were all too low, indicating no practical significance. There was no correlation between patients’ salivary cortisol levels 40 minutes after the task and the PANSS total scores, negative subscale scores, positive subscale scores, or general psychopathological subscale scales. None of the PANSS scales were correlated with the patients’ NA scores.

### Rigor

We performed re-analyses including data from the 13 participants with extreme values that were previously excluded, and this did not change the outcomes.

## Discussion

### Cortisol and schizophrenia

Changes in saliva cortisol levels before and after stressor exposure represent the function of the cortisol stress response and HPA axis. The previous stress-vulnerability model theoretically proposed the relationship between HPA axis function and schizophrenia^1–4^, which has been verified in several studies^12^. In this study, the existence of interactions between time and diagnosis showed that the trends in the cortisol level changes of the two groups differed greatly as the psychological stress challenge task progressed. The patients’ cortisol stress response was not obvious: although it tended to first rise and then fall, the change was not significant (*p* > 0.05). The overall response was flattened, which was similar to the blunted CAR previously reported in patients with schizophrenia^18–20,22^. The changes in salivary cortisol levels differed greatly between the patient group and the control individuals; in the control group, the level was clearly high before the task began and then gradually decreased, while it did not change significantly throughout the course of the experiment in patients with schizophrenia. The difference in cortisol stress response between patients with schizophrenia and healthy controls was obvious, confirming the hypothesis that patients with schizophrenia had HPA axis dysfunction.

### Cortisol, stress, and distress intolerance

We chose to measure cortisol stress response, as it better reflects the pathological process than the baseline cortisol level does^12^.

For some participants, the imminent participation in the test may have caused anticipatory anxiety. Although our study did not take samples at the same time another day for comparison, a previous study performed this comparison, confirming the existence of the expected anxiety^30^. We found that the pre-task cortisol levels among the controls were significantly higher than the post-task cortisol levels, which is inconsistent with the findings from a previous study in an American population^16^. As we controlled the same initial experimental conditions for all participants, such as experimenting at the same time in the afternoon and avoiding strenuous exercise, eating, drinking, smoking, brushing teeth, and other actions that may affect cortisol test values before the experiment, we believe that these cortisol levels represented stress levels. As there was no evidence that stressful psychological tasks decreased salivary cortisol, we inferred that the cortisol curve of the control group decreased owing to the pre-task cortisol elevation. Elevated cortisol levels suggest that the healthy controls already had significant sensations of stress before the task began; hence, the HPA axis could not respond to a new stressor (the task) because the feedback mechanism was already in action. Considering that previous studies were conducted in the Western parts of the world, some differences may have been due to our participants being from a Han Chinese population. Many studies have revealed racial differences in cortisol levels^36–39^, and one study also found that people of different ethnicities have different responses to psychological support under pressure^40^, suggesting that cortisol and stress-related findings may differ between different ethnic groups. However, no study has specifically investigated the effect of racial differences on expected anxiety or cortisol levels yet; hence, this point warrants further study.

The influence of culturally related psychological factors is another possible reason for the differences. Studies have shown that differences between the Eastern and Western cultures affect people’s attitudes toward examinations. In some cultural atmospheres that emphasize examinations, test anxiety is more obvious and common^41,42^; China has such a cultural atmosphere, and compared with that of participants in previous Western studies^16,30^, our participants had a low task withdrawal rate, which may have also reflected this cultural atmosphere. After learning they would take a test, the healthy controls might have had higher expectations for their performance, which could also have caused severe anticipation anxiety. In contrast, the patients felt less stressed before the task, which is consistent with the apathy and decreased self-expectation seen in schizophrenia^43^.

Whether due to anticipatory anxiety or the psychological stress challenge task, cortisol levels in the control group changed significantly throughout the test, while cortisol levels in the patient group did not. Here, we propose two conjectures that might explain this phenomenon. One reason may be that cortisol levels in the patient group were less responsive to stressors, which is in line with the results of previous studies^17^. A previous meta-analysis also showed that stress reactivity was blunted in schizophrenia; however, the number of papers included in the analysis was low; therefore, the conclusions remain to be validated^12^. Additionally, the patient group may have had lower basal cortisol levels and, similar to those of the control individuals, their cortisol levels were already elevated prior to the task; therefore, we captured a high range of their own cortisol levels. As their cortisol levels failed to decrease after the stressor was removed, indicating that the response of their HPA axis may have been prolonged or the negative feedback mechanism was damaged, rendering them unable to escape from stress. This is in line with the findings of earlier studies, that showed a significantly prolonged cortisol stress response among patients with schizophrenia^16,31^. Both of these possibilities suggest that the patients have abnormal HPA axis function and the patients with schizophrenia may have one or both of these conditions. However, our current study lacks baseline data during the calm state for the participants; therefore, we can only make conjectures, as it is not possible to verify whether the above possibilities are correct. A more rigorous study design is needed to test these possibilities; future studies should consider increasing the baseline sampling and improving the study design.

Among the patients, the trends of changes in cortisol levels in distress tolerance and distress-intolerance patients were different. Among patients with distress intolerance, there was an upward trend in cortisol levels during the task, followed by a drastic drop after the task. This may indicate that these patients felt that the pressure during the task greatly increased and exceeded their tolerance threshold, leading them to opt-out. When they decided to give up, most of the stress disappeared, and cortisol levels dropped rapidly. However, patients with distress tolerance may have been insensitive to the stress caused by the task, leading to indifference towards completing the task and smoother cortisol level curves. However, as these results were not significant, which may be related to the small sample size of the distress intolerance group, it is only speculation. These conjectures warrant further verification by expanding the sample size.

### Clinical symptoms and other indicators

We found that cortisol stress response was not significantly correlated to patient symptoms, nor was it correlated with negative emotion scores before the task. Education level and smoking status had no significant effect on cortisol stress response, which is consistent with previous reports^16,17^.

Although there are few studies on cortisol stress response in patients with schizophrenia, there are some studies on other cortisol indicators with inconsistent results. Most of them found no significant correlation between cortisol and symptoms^44–46^, with similar findings for CHR patients^13,23^. However, a study of treatment-resistant patients with schizophrenia found that patients’ serum cortisol levels were significantly reduced in the morning after treatment, and, although the correlation was weak, it was related to improvement in PANSS negative symptoms^47^. One study also found that, in chronic schizophrenia, cortisol levels were weakly correlated with the PANSS negative-symptom subscale scores^48^. Another study found that, after treatment, the increase in cortisol levels during the day in patients with schizophrenia was relieved, but the blunted CAR did not change^22^. These differences may have been due to variation in research methods. Previous studies showed that symptoms may be related to cortisol levels within a certain period, reflecting the daily function of the HPA axis, while cortisol stress response represents the mobilization and coping ability of the HPA axis (similar to the CAR). This suggests that HPA axis mobilization and abnormal coping ability may be a stable biological indicator of disease that do not fluctuate with the severity of symptoms and are less affected by other factors. Therefore, it has clinical predictive significance and may even become an endophenotype.

We found that the patients with distress intolerance had more severe clinical symptoms, while distress intolerance was very rare in the control group, indicating that distress intolerance may represent a state that is more deviated from the healthy state. However, whether distress intolerance is the result of symptoms or one of the factors that cause obvious symptoms remains to be studied.

A previous study found poorer memory and executive functioning in people with schizophrenia^9^. We found that the error rate of the MTPT in the patient group was significantly higher than that in the control group, which we speculate may be related to the impaired cognitive function in patients with schizophrenia. However, this study did not measure cognitive function, making it difficult to draw any conclusions.

### Negative emotions

The differences in NA scores between patients and controls are understandable. The psychological stress challenge task is challenging, requires concentration and patience, and plays annoying stimuli in response to a mistake—a process that is obviously unpleasant. As expected, the NA scores of the controls increased as the task progressed; however, patients with schizophrenia were already experiencing negative emotions before starting the task. The feeling remained almost unchanged during the task, indicating that patients with schizophrenia were less satisfied with their daily life or less satisfied with their participation in the test—the content of the task was not their focus. Although there are no other studies that used the same psychological stress tasks and mood assessment scales as ours, studies that used the Trier social stress test similarly found that patients with schizophrenia showed a blunted physiologic reactivity to psychological stress and no significant changes in mood^49,50^.

### Strengths and limitations

To our knowledge, no large-sample study was previously performed to examine the correlation between cortisol stress response and schizophrenia in a Chinese population. Possible racial differences make our research more meaningful. We collected data of more than 160 cases, with is a larger sample size than that of previous studies.

Cortisol levels may also be affected by other factors, such as alcohol intake or body weight, which were not considered in our study. Because the main focus of our study was the change in cortisol levels under stress, there was a self-control effect; hence, the aforementioned factors would have had a limited impact on the interpretation of the results. Naturally, if complete information could be obtained from the participants, it would help improve the rigor of our conclusions. Cross-sectional studies cannot rule out differences in individual sensitivity to cortisol. Although we included self-controls and a relatively large sample size—minimizing the effect of differences in sensitivity on the results—more information on sensitivity is needed.

Age and sex have some complex non-linear effects on cortisol^51^. We could not perform a more detailed subgrouping owing to the limited sample size, but our patients and controls were age- and sex-matched to reduce the influence of these variables. We would like to perform a finer grouping of patients, including the effects of disease stage and medication. A recent review suggests that long-term use of a single antipsychotic may indirectly affect cortisol levels through actions on the prefrontal cortex, hippocampus, and inflammatory factors^52^. However, the sample size was insufficient for more detailed grouping, which should be addressed in future studies.

The lack of baseline cortisol measurement is a major limitation of the study design that affected the conclusions. The participants were ready to start the task before the first saliva collection; therefore, they were not in a state of complete rest. A more complete assessment of a participant’s stress state can be achieved by measuring resting cortisol levels over the same period on a different day along with measuring blood pressure, heart rate, and markers of sympathetic activation, such as salivary alpha-amylase activity, to increase the reliability of the results.

Data have been obtained from cross-sectional studies, but disease and treatment conditions may progress or change. Therefore, long-term follow-up data are required to confirm the stability of cortisol stress response in individual treatment.

## Conclusions

Schizophrenia is a major focus of psychiatry research, and the identification of biological indicators is important for clinical diagnosis. We found significant differences in cortisol level trends during stressful tasks between patients with schizophrenia and healthy controls. The mechanism of this difference is yet to be determined; however, our results can inform future follow-up studies exploring the mechanism of abnormal cortisol stress response and its use, alone or in combination, as a possible biomarker for schizophrenia.

## Supplementary Information

Supplementary Information.

## Data Availability

All data generated or analysed during this study are included in this published article and its supplementary information files.
